# Stacking change in MoS_2_ bilayers induced by interstitial Mo impurities

**DOI:** 10.1038/s41598-018-20289-1

**Published:** 2018-02-01

**Authors:** Natalia Cortés, Luis Rosales, Pedro A. Orellana, Andrés Ayuela, Jhon W. González

**Affiliations:** 1 Universidad Técnica Federico Santa María, Departamento de Física, Valparaíso, Casilla 110V Chile; 2 Centro de Física de Materiales (CSIC-UPV/EHU)-Material Physics Center (MPC), Donostia International Physics Center (DIPC), Departamento de Física de Materiales, San Sebastián, 20018 Spain

## Abstract

We use a theoretical approach to reveal the electronic and structural properties of molybdenum impurities between MoS_2_ bilayers. We find that interstitial Mo impurities are able to reverse the well-known stability order of the pristine bilayer, because the most stable form of stacking changes from AA’ (undoped) into AB’ (doped). The occurrence of Mo impurities in different positions shows their split electronic levels in the energy gap, following octahedral and tetrahedral crystal fields. The energy stability is related to the accommodation of Mo impurities compacted in hollow sites between layers. Other less stable configurations for Mo dopants have larger interlayer distances and band gaps than those for the most stable stacking. Our findings suggest possible applications such as exciton trapping in layers around impurities, and the control of bilayer stacking by Mo impurities in the growth process.

## Introduction

The recent isolation of new 2D materials, such as hexagonal boron nitride (h-BN)^1^, black phosphorus^2^, and particularly transition metal dichalcogenides (TMDCs)^3^ have attracted considerable attention thanks to their interesting physical, chemical, electronic, optical, and mechanical properties^4–11^. Among TMDCs, MoS_2_ is being used as prototype in several different applications, such as photovoltaic cells, photocatalysts, electronic nanodevices, and energy storage and conversion materials^12^. In structural terms, a layer of MoS_2_ has Mo centers coordinated with six sulfur ligands in a trigonal prismatic arrangement^13,14^, following a hexagonal lattice of alternating Mo and S atoms, as seen from above. TMDCs nanostructures can then be produced by stacking several MoS_2_ layers through weak van der Waals interactions. It is noteworthy that the number of layers and their stacking arrangement largely modify the electronic properties of the MoS_2_ semiconductor^15–19^. For instance, the MoS_2_ monolayer shows a direct band gap, compared with the indirect band gap of MoS_2_ bulk^20–23^. The stacking in the MoS_2_ bilayer alters the band gap, which can then be engineered not only by strain^24,25^, but also by sliding^26^ and twisting the MoS_2_ layers^27^. Thus, the control of the MoS_2_ bilayer stacking is relevant when considering different applications in future devices^28^.

The electronic and magnetic properties of MoS_2_ monolayers and bilayers are also tuned by defects, such as vacancies and adatoms^29–31^. Most of the adatom impurities considered within MoS_2_ layers are in groups IA and VIIA, or transition metal atoms. The adatoms in the interlayer space have interesting effects, such as *n*- or *p*-type doping, induced magnetic moments^32–34^, and structural phase transitions^35^. Although some early photoelectron spectroscopy experiments appeared to show Mo atoms embedded between MoS_2_ layers^36^, current experiments using low-temperature scanning tunneling microscopy (STM) show Mo impurities between bilayers^37^. However, the effects of intrinsic Mo impurity atoms on the electronic and structural properties of MoS_2_ bilayers with different stackings are still largely unexplored.

We present a theoretical study of the structural and electronic properties of the MoS_2_ bilayer considering Mo atoms as intrinsic impurities, placed at different positions within the interlayer region in a diluted regime. Using density functional with van der Waals calculations from first principles, we relax the structures and study their electronic properties. We find that the intrinsic Mo impurities in the interlayer region produce some interesting behaviors, namely (i) a change in the stability order with respect to the pristine bilayer, energetically favoring the AB’ stacking over the AA’, (ii) impurity states in the band gap region, and (iii) an increase in the distance between layers. The structural and electronic modifications induced by the impurities could be employed as electron and exciton-like traps^38,39^, and the change in stacking produced by the Mo impurities could also be useful to fine-tune the stacking during the growing process^19,27^.

## Model

**Figure 1:**
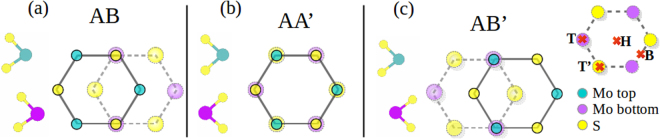
Stackings of MoS_2_ bilayers considered with low-energy: (**a**) AB, (**b**) AA’ and (**c**) AB’. Mo atoms are shown as cyan spheres, and sulfur atoms are shown as yellow spheres. Inset shows the Mo impurity sites relative to the bottom layer in B (bridge), H (hollow), T (top over Mo) and T’ (top over S).

We first consider the most stable stackings of the pristine MoS_2_ bilayers, namely the 2H and the 3R phases^28,40,41^. These natural phases have Mo atoms in a trigonal prismatic coordination, and the MoS_2_ layers in different stacking orders. The bilayer in the 2H-phase has inversion symmetry^7,22^, and shows the AA’ and AB’ stacking related by rotating the MoS_2_ layers. In the AA’ stacking, the hexagons in each layer are superposed in such a way that the molybdenum atoms of the bottom layer are located just below the sulfur atoms in the top layer, and vice-versa. For AB’ stacking the hexagons in each layer are shifted with the sulfur atoms of the bottom layer beneath the hollow sites of top layer, and the molybdenum atoms in the top layer over the molybdenum atoms in the bottom layer^18^, as shown schematically in Fig. 1. In our calculations we found an energy difference between these stackings of E_AA′_ − E_AB′_ = 2.6 meV per atom, in good agreement with previous DFT calculations^18,19,42^. The AB stacking with no rotation between two layers belongs to another kind of phase, the 3R-phase. The up layer slides on the bottom layer in the armchair direction so that some Mo and S atoms in different layers match. Our results for the stability order of the pristine bilayers show that AB stacking is the most stable, nearly degenerated with the AA’ stacking with a total energy of 0.7 meV per atom, followed by the AB’ stacking with 3.3 meV per atom. Note that depending on the details of different calculations the ground energy stacking can exchanging between AB and AA’^43^, and the AB’ stacking remains as the third state in stability.^18,19,42,43^.

We then include Mo atoms as intrinsic impurities within the interlayer region for MoS_2_ bilayers at different inequivalent positions, as shown in Fig. 1. The Mo impurity is labeled as Mo_imp_. The initial absorption sites for Mo_imp_ within the MoS_2_ bilayer are assumed to follow Mo absorption sites as in MoS_2_ monolayers^44,45^. The Mo impurity position is then labeled relative to the bottom layer in B (bridge), H (hollow), T (top over Mo) and T’ (top over S). The MoS_2_ bilayer structures with the impurity in the interlayer region are fully relaxed, to allow the optimized lattice parameters and atomic coordinates to be obtained. More detailed information on the relaxed geometries is included in the Supplemental Material. The binding energy can then be calculated using *E*_binding_ = *E*_Total_ − *E*_bilayer_ − *E*_imp_, where *E*_Total_ is the total energy of the MoS_2_ bilayer with the impurity, *E*_bilayer_ is the energy of the corresponding final pristine MoS_2_ bilayer (either AA’ or AB’), and *E*_imp_ is the energy for the isolated Mo_imp_ atom.

## Results

### Energy and Geometry

Because the most stable configuration with Mo_imp_ belongs to the 2H-phase, in the main text we discuss the impurity properties between bilayers focusing on the AA’ and AB’ stacking. The discussion on the 3R-phase is reported as Supplemental Material.

**Figure 2:**
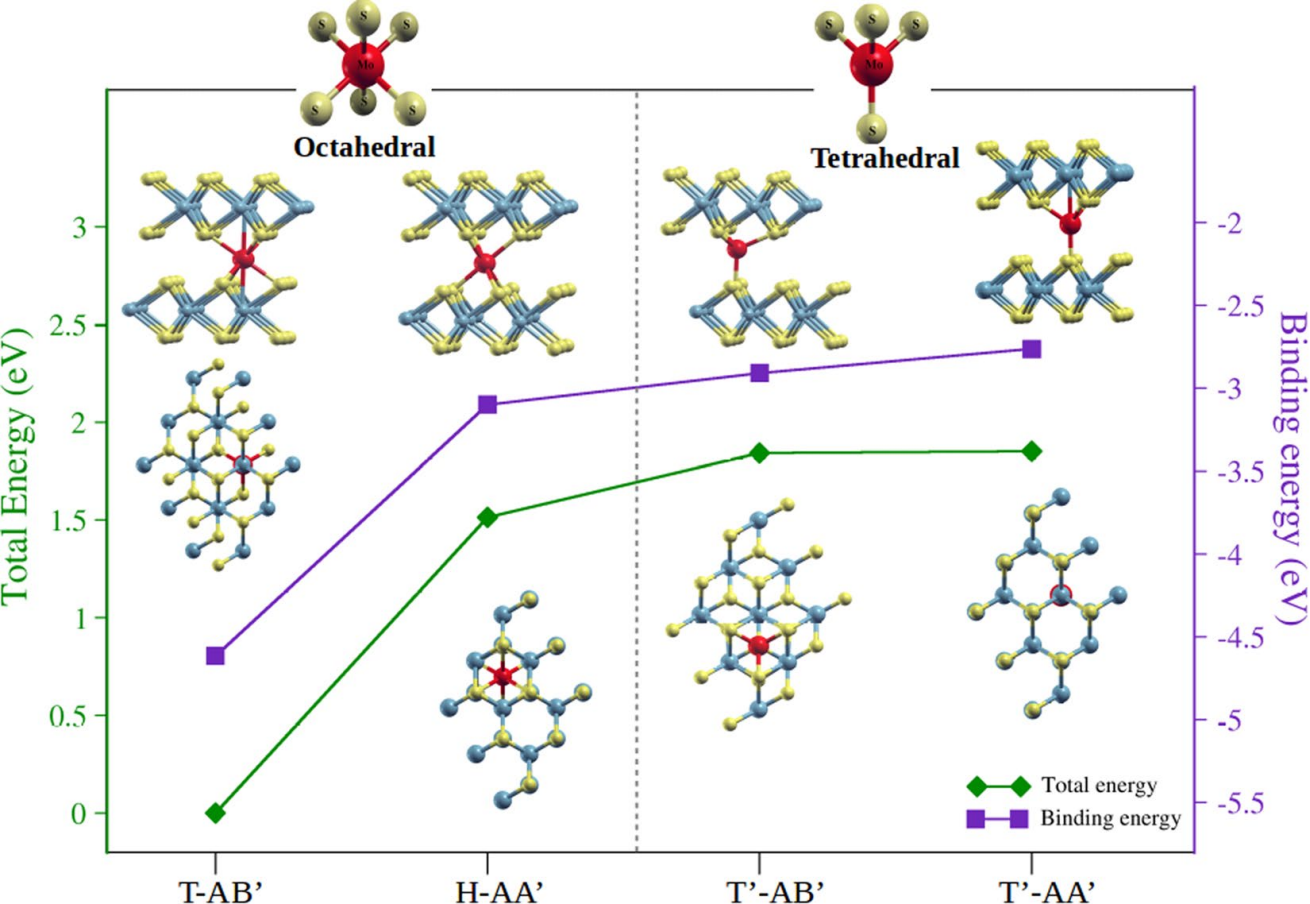
Total and binding energy as function of the structural configurations of Mo impurities within the MoS_2_ bilayer. The red spheres indicate the Mo impurity in each configuration. The relaxed structures are included, grouped in octahedral (T-AB’ and H-AA’) and tetrahedral (T’-AB’ and T’-AA’) structures of sulfur atoms around Mo impurities. The zero energy point is set for the most energetically favorable structure, namely the T-AB’ configuration. Figure prepared using XCrySDen^46^.

Figure 2 shows the total and binding energies for the different 2H stacking and impurity positions. The binding energies are negative, which indicates that the Mo impurity atoms are indeed adsorbed in the interlayer region of the MoS_2_ bilayer. The binding and total energies exhibit the same trend in terms of stability. The results in increasing order of stability show that in the presence of the interlayer Mo_imp_, the T-AB’ bilayer configuration is the most energetically favorable. This configuration has AB’ stacking with the Mo_imp_ superposed with two Mo atoms as seen from above. Note that the T-AB’ configuration is reached from the input that has the Mo_imp_ placed at the bridge (B) position in the AA’ stacking.

The next most favorable configuration is H-AA’, with the Mo_imp_ in the hollow position, in which the bilayer structure maintains the AA’ stacking. The H-AA’ case is less stable than T-AB’ by about 1.5 eV. On the right hand side, the configurations labeled T’-AB’ for AB’ stacking and T’-AA’ for AA’ stacking are energetically close, and the least stable.

We classify the relaxed configurations according to how the Mo_imp_ is related structurally to its neighboring sulfur atoms^47,48^. The T-AB’ and H-AA’ configurations form octahedral sites around the Mo_imp_. These configurations have a coordination number of six, corresponding to the six neighboring sulfur atoms. The T’-AA’ and T’-AB’ configurations for the Mo_imp_ form a tetrahedral structure with a coordination number of four, therefore, the sulfur atoms in the top and bottom layers enclose a tetrahedral site for the Mo_imp_. These octahedral and tetrahedral environments are shown schematically at the top of Fig. 2. It is noteworthy that regardless of the final stacking, the octahedral configurations are the most stable.

### Electronic Properties

**Figure 3:**
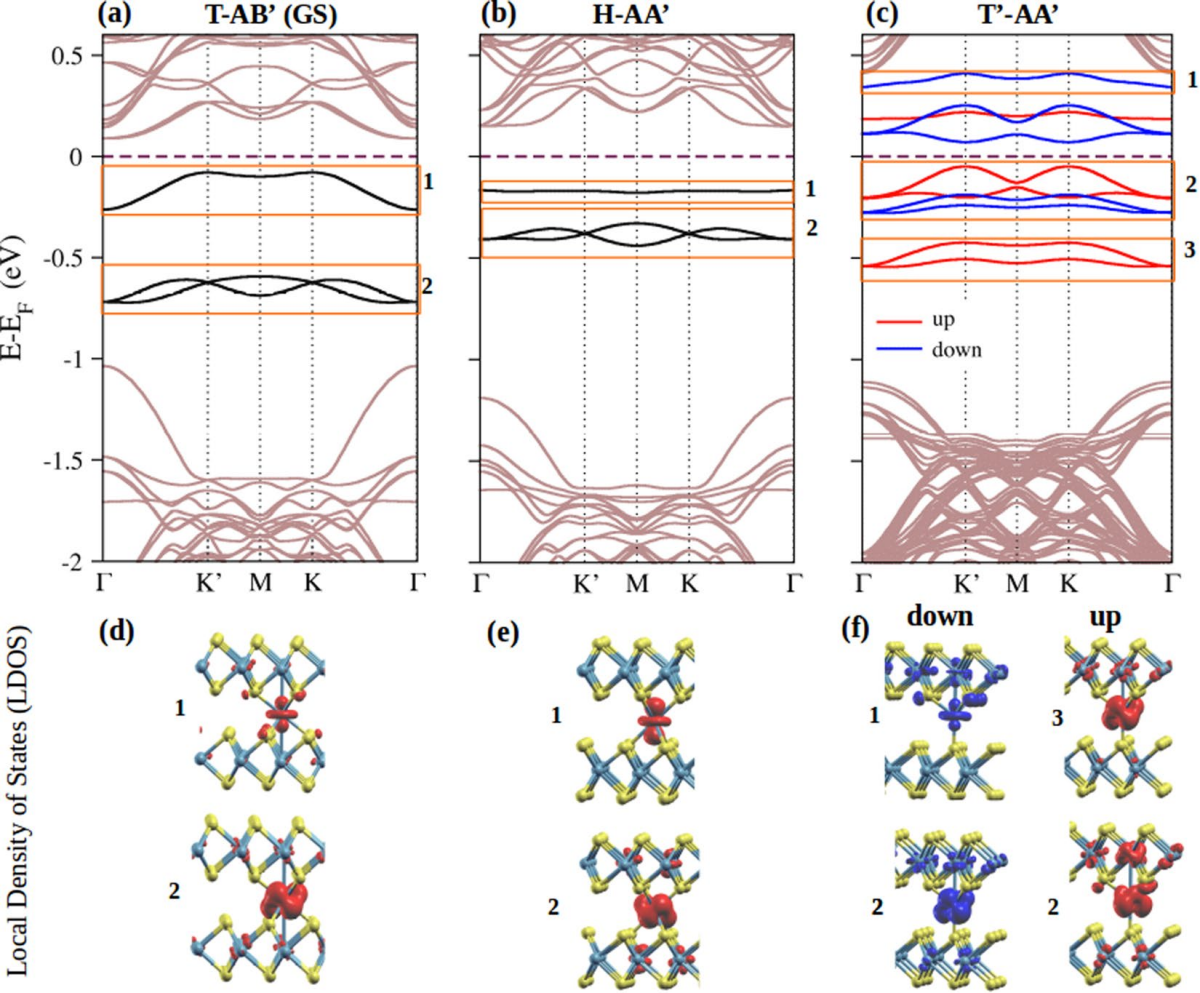
(**a**–**c**) Band structures for the given configurations. The Fermi energy is set to 0 eV. Orange rectangles enclose the Mo_imp_ bands separated into several different energy regions, labeled as **1**, **2** and **3**. (**d**–**f**) Local density of states (LDOS) projected in space for the Mo_imp_ bands in the band gap region of the MoS_2_ bilayer. Figure prepared using XCrySDen^46^.

Results showing the band structures and the local density of states (LDOS) projected in space, for some of the considered configurations, are presented in Fig. 3. We focus on the impurity in-gap states near the Fermi energy introduced by the Mo_imp_ atom, indicated by the areas enclosed by orange rectangles. The band structures of the most stable structures show three distinctive in-gap states, joined in two groups, labeled as regions **1** and **2** with degeneracies of one and two, respectively. Although the Mo_imp_ in these two configurations have an octahedral sulfur environment, the in-gap bands present slightly different dispersive behavior. In region **1**, the band for the T-AB’ case is more dispersive than the corresponding band for the H-AA’ configuration, which is almost flat. The states in region **1** mainly have ${{\rm{d}}}_{{z}^{2}}$ orbital character, as shown by the LDOS in panels (d) and (e). Note that in region **1**, the surrounding region of the impurity for the T-AB’ case has some hybridization with bilayer orbitals, which is not observed for the H-AA’ case. In region **2** of the T-AB’ case, there are two energy bands which are mainly non-bonding Mo_imp_ d orbitals with the neighboring sulfur atoms, as shown by the LDOS in panels (d) and (e). By comparing the T-AB’ and H-AA’ configurations, the stability order can be associated with the widening of the bands in regions **1** and **2** and to the displacement of the bands in region **2** to lower energies in the T-AB’ configuration.

The band structures in the T-AB’ and H-AA’ configurations are spin-compensated and related to the impurity in an octahedral sulfur environment. We now focus on the T’-AA’ and T’-AB’ cases. These two configurations have similar energy band structures and are found to have close energies. Both systems exhibit spin polarized behavior with a total magnetic moment of 2 *μ*_*B*_, which is determined by a similar Mo_imp_ tetrahedral environment. In particular for the T’-AA’ configuration, the spin up and spin down components of the spatial resolved LDOS are shown in Fig. 3 panel (f). The state in region **1** has a ${{\rm{d}}}_{{z}^{2}}$ orbital character; however, it is above the Fermi level. The LDOS of the lower impurity states, the up states in region **3** and the spin down component in region **2** are nearly equal, depleting the LDOS in the Mo_imp_-S bond direction as in the T-AB’ case. However, the states responsible for the spin polarization in the T’-AB’ and T’-AA’ configurations are the spin up d-orbitals in region **2**, which are along the Mo_imp_-S bonds.

We also find that the LDOS is localized not only on the Mo_imp_, but also on one of the MoS_2_ layers. This layer asymmetry indicates that doping by electrons or holes could spatially differentiate between the two layers in the MoS_2_ bilayer, a finding that could be of use in optoelectronic applications^22,49^.

**Figure 4:**
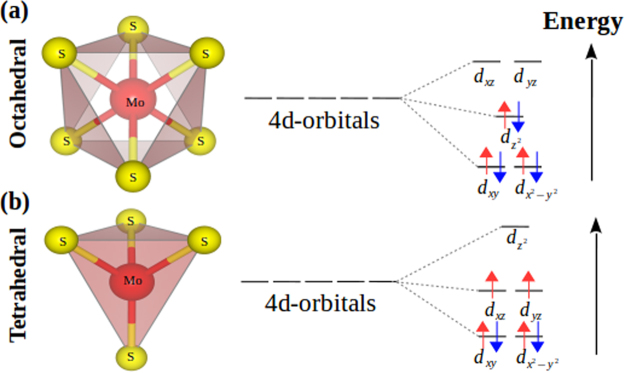
Ligand field splitting for Mo_imp_ d-orbitals produced by the neighboring sulfur atoms in (**a**) octahedral and (**b**) tetrahedral environments. Figure prepared using VESTA^50^.

The electronic structure for the impurity level states is best understood using crystal field theory. We analyze the ligand field splitting for the Mo_imp_ d-orbitals produced by the interactions with the sulfur ligands for the octahedral and tetrahedral sites. The bonding and non-bonding interactions of d-orbitals for octahedral and tetrahedral sites are in agreement with the energy level scheme shown in Fig. 4. We consider the *z*-axis perpendicular to the layers and the *x* and *y* axes in the in-plane layer. In the octahedral environment for the T-AB’ and H-AA’ configurations, the sulfur ligands overlap less with the in-plane d_*xy*_ and ${{\rm{d}}}_{{x}^{2}-{y}^{2}}$ orbitals, these orbitals are therefore non-bonding and have the lowest energy. The ${{\rm{d}}}_{{z}^{2}}$ orbital remains non-bonding at an intermediate energy, interacting less with the sulfur atoms. We next find that the d_*xz*_ and d_*yz*_ orbitals are more strongly directed and interact with the sulfur atoms along Mo_imp_-S bonds, lying at higher energies, as is showed in Fig. 4(a). In the case of tetrahedral environment for T’-AB’ and T’-AA’, shown in Fig. 4(b), the d_*xy*_ and ${{\rm{d}}}_{{x}^{2}-{y}^{2}}$ orbitals behave similarly to the octahedral structure; however, the d_*xz*_ and d_*yz*_ orbitals exchange roles with the ${{\rm{d}}}_{{z}^{2}}$ orbital. Thus, the ${{\rm{d}}}_{{z}^{2}}$ orbital in the tetrahedral environment interacts more with the sulfur ligands increasing its energy, as shown for region **1** of Fig. 3(c) and (f) in the T’-AA’ case. The level filling help to explain why the tetrahedral cases have spin polarization, and a total magnetization of 2 *μ*_*B*_, as shown by the arrow counting.

Molybdenum impurities in the 3R-phase follow the same general trends as the previously discussed for the 2H stackings. The octahedral Mo environment is the most stable and the tetrahedral environment is less stable and presents a non-zero magnetic moment. A more detailed discussion of the stacking configurations for the 3R-phase with Mo impurities is included in Supplemental Material.

The main effect in our calculations is the interaction of the Mo impurity with the MoS_2_ layers in the order of eVs. The effect of spin-orbit coupling (SOC) in the stacking stability is expected to be minimal although it would split bands near the Fermi level^7,51^. Since the spin-orbit interaction is particularly noticeable in materials without inversion symmetry^52^, we can expect the following effects. On the one hand, the pristine bilayers in the 2H-phase presents point-center inversion symmetry, and the band structure remains spin degenerate even in the presence of SOC^14,40^. SOC can thus change the pristine bilayer bands in the 3R-phase because the lack of inversion symmetry would breaks the spin degeneracy and could lead to valley dependent spin polarization^53^. On the other hand, we can expect splitting of the Mo_imp_-bands due to the presence of spin-orbit and the lack of inversion symmetry in the tetrahedral configurations (in Fig. 4). However, the impurity in the tetrahedral arrangement is not the most stable configuration by an energy difference of 1.7 eV, much large than values typically associated to the spin-orbit coupling energies.

We now consider the gap changes in the bulk bands, shown in light-color in Fig. 3, and induced by the Mo_imp_. It is important to mention that the indirect gap along Γ − *K* direction of 1 × 1 unit cell becomes a direct band gap at the Γ-point due to the k-space folding of the large 3 × 3 unit cell. Our calculated pristine bands are in agreement with the literature, and are included in Supplemental Material. It is well known that band gaps calculated using GGA/LDA infra-estimate the values produced in experiments, so we discuss differences in magnitude gaps. The layer-gap is indicated by the energy difference between the HOMO-LUMO bulk bands at the Γ-point. The energy gaps of the pristine MoS_2_ layers are correlated with the interlayer distances. We check that the gap and distances for the AB’ pristine stacking are 0.09 eV smaller and 0.03 Å shorter than the values for the AA’ pristine case. The layer-gaps and the interlayer separation including Mo_imp_ show larger values in comparison with the pristine cases. Among the Mo doped systems, the most stable T-AB’ case has the smaller layer-gap and the shortest interlayer separation. The layer-gap is 0.2 eV above the AB’-pristine, and the interlayer separation is 0.03 Å larger than that of AB’-pristine. The layer-gap values for the H-AA’ and the T’-AB’ (T’-AA’) increase from the T-AB’ case by 0.2 eV and by 0.5 eV respectively. These gap differences are somewhat correlated with the difference between layer-layer distances, ∼0.5 Å, between the T-AB’ and H-AA’ cases, a value that increases up to 0.7 Å for the T’-AB’ and T’-AA’ configurations. The increase in the layer-band-gap with interlayer distance is explained by a weaker interlayer coupling. Detailed information is provided in the Supplemental Material.

The interlayer distances in the proximity of the impurity are between 0.1 and 0.16 Å larger than those far from it, which indicates the role of local strain. Furthermore, experiments prove than the band gap of bilayer TMDCs can be controlled by strain^39,54,55^. We propose that the electronic and structural modifications around the impurity could be used in a similar way to electronic confinement for embedded quantum dots. Current experimental techniques employing cross-sectional scanning transmission electron microscope analysis in encapsulated TMDC materials can provide evidence of impurity species being trapped in the interstitial region^56^. This effect thus has potential applications for optoelectronic devices as exciton traps around Mo-doped bilayers. A number of different experimental techniques can be used to corroborate our theoretical predictions, for instance angle-resolved photoemission spectroscopy and cross-sectional scanning transmission electron microscope analysis^56,57^.

### Stacking change

Another possible implication of our results is that transition metal ions could be used to engineer the stacking between TMDC bilayers and to tune their electronic properties. In the T-AB’ and H-AA’ configurations, the Mo_imp_ is located within sulfur ligands forming octahedral sites. In these two configurations, the Mo_imp_ presents structural differences in the relative position respect to the nearest Mo atoms, belonging to the top and bottom MoS_2_ layers. The Mo_imp_ bonding produces a stacking change in T-AB’ related to the total energy gain. The interlayer Mo-Mo distance around the impurity is smaller around 0.1 Å than the interlayer distance away from the impurity. The shorter distance promotes the hybridization of the impurity states with the layer states increasing the dispersion of the in-gap impurity states.

**Figure 5:**
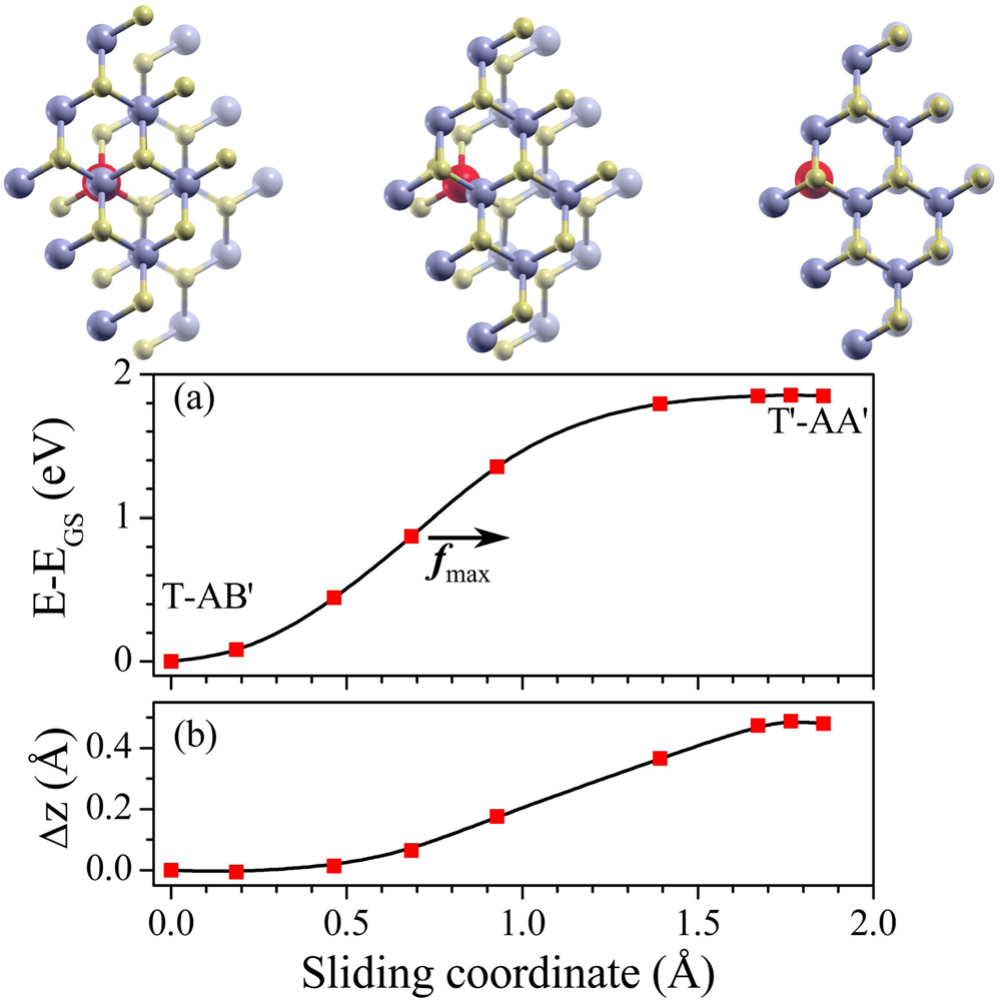
(**a**) Energy profile and (**b**) interlayer distance (Δ_*z*_) by sliding between the 2H stackings. The sliding coordinate goes along the armchair direction. Full lines are just guiding the eyes. Energies and distances shown are referenced with respect to to the most stable configuration.

A scheme for the stacking change is shown in Fig. 5. Starting from the AB’ stacking, the AA’ stacking is found by shifting a layer in the armchair direction. The maximum sliding coordinate corresponds to the distance of the Mo-atom to the center of the hexagon, *a*_0_ ≈ 1.86 Å. The energy profile along the sliding coordinate is calculated shifting the top layer, then fixing the in-plane coordinates for the Mo_imp_ and a MoS_2_ unit far from the impurity and relaxing. Figure 5(a) shows the total energy along the above discussed sliding route. Away from the T-AB’ configuration the energy increases smoothly passing through an energy maximum before reaching the final T’-AA’ configuration. The maximum energy is found for a displacement of 1.76 Å (≈0.95 *a*_0_) being 5 meV above the T’-AA’ value. It is noteworthy that the total energy as a function the sliding coordinate shows an inflexion point corresponding to the point where the slip force is maximum. That point corresponds to a displacement of 0.69 Å (≈0.37 *a*_0_) in the armchair direction. The slipping force calculated as the derivative of the total energy as a function of displacement shows a maximum force around 2.1 eV/Å, similar to the forces calculated with DFT.

The Mo-Mo interlayer distance denoted as Δ_*z*_ follows a similar trend as the total energy (Fig. 5 (b)). For the T’-AA’ configuration, the interlayer distance is 0.48 Å larger than the value for the most stable configuration T-AB’. The Δ_z_ maximum is found for a displacement of 1.76 Å ≈ 0.95 *a*_0_ where the Δ_z_ separation is 10 mÅ higher than in T’-AA’ . The maximum force and change in z along sliding seems interesting parameters to describe tribology between MoS_2_ layers^26,58^.

## Conclusion

We studied the structural and electronic properties of a MoS_2_ bilayer with intrinsic Mo impurities within the interlayer region.We find that the most stable configuration is T-AB’, with an energy gain above the van der Waals interaction because the Mo impurity levels strongly hybridize with the nearest atoms. A change in the stacking stability order from AA’ to AB’ is observed to be induced by impurities, with the corresponding change in energy gap. Thus, it is possible to engineer the stacking between TMDC bilayers during the growth process, enabling their electronic properties to be fine-tuned. The states and deformations induced by impurities could also be used for electronic confinement applications in optoelectronic devices, based on exciton/electron trapping.

## Simulation Details

The MoS_2_ bilayer systems was described using density functional of van der Waals (vdW-DF) calculations with the SIESTA method^59^. To describe the core electrons, we consider norm-conserving relativistic ab-initio pseudopotentials in the Troullier Martins form^60^, including nonlinear core corrections for inner d-electrons^61^. The exchange and correlation energy are calculated by the non-local vdW-DF, using the parametrization proposed by Dion *et al*.^62^, taking into account the exchange energy modification included by Cooper (C09)^63^. In the C09 parametrization, the long-range dispersion effects are included as a perturbation to the local-density approximation correlation term. The van der Waals parameterization was chosen after comparing the band structure of the AA’ system for the 1 × 1 unit cell with the existing literature^22^, in particular the presence of an indirect band gap between the Γ and K points. Note that the bottom of the conduction band can move away from point K-point depending on the chosen van der Waals functional.

To include impurities, the structures of the MoS_2_ bilayers was extended to a 3 × 3 MoS_2_ supercell, using periodic boundary conditions. We are dealing with a diluted regime corresponding to 1 impurity every 54 atoms in the unit cell, around 2% doping reasonable in experimental setups. Basis set is double-*ζ* polarized (DZP) with numerical atomic orbital with an energy shift of 30 meV, converged to have an extended basis good to describe long van der Waals bonds. The mesh cutoff energy for the integration grid was well converged using 230 Ry. A *k*-grid of 10 × 10 × 1 Monkhorst-Pack is used to sample the Brillouin zone. A vacuum region in the *z*-direction of at least 20 Å avoids interactions with periodic images. The structures were relaxed until the force in each atom was less than 10^−2^ eV/Å. Further technical details are included in Supplemental Material.

## Electronic supplementary material

Supplemental Material
