# Supplementary material for: Stacking change in MoS2 bilayers induced by interstitial Mo impurities
Source: Sci Rep. 2018 Feb 1;8:2143. doi: 10.1038/s41598-018-20289-1 (PMC5794788; doi:10.1038/s41598-018-20289-1)
Supplement: Supplementary file 1 — Supplemental Material [file 41598_2018_20289_MOESM1_ESM.pdf]

Supplemental material: Stacking change in MoS_2 bilayers induced by interstitial Mo impurities

Natalia Cortés[1,*], Luis Rosales[1], Pedro A. Orellana[1], Andrés. Ayuela[2], and Jhon W. González[2]

[1]Universidad Técnica Federico Santa María, Departamento de Física, Valparaíso, Casilla 110V, Chile
[2]Centro de Física de Materiales (CSIC-UPV/EHU)-Material Physics Center (MPC), Donostia International Physics Center (DIPC), Departamento de Física de Materiales, San Sebastián, 20018, Spain
[*]natalia.cortesm@usm.cl

ABSTRACT

In this Supplemental Material, we report some calculations and parameters for the MoS_2 pristine bilayers and MoS_2-impurity bilayers in the 2H-phase (with AA' and AB' stacking) and 3R-phase (in AB stacking).

SI Pristine band-structures

Fig. S1 shows the band structures for the most stable MoS_2 pristine bilayers in 1×1 unit cell. In the MoS_2 bilayers with impurities, we use in-plane lattice vectors three times larger than the basic MoS_2 unit cell. Thus, the $\Gamma - K$ indirect gap of the 1×1 unit cell shown in Fig. S1 becomes a direct band gap at the Γ-point because of k-space folding.

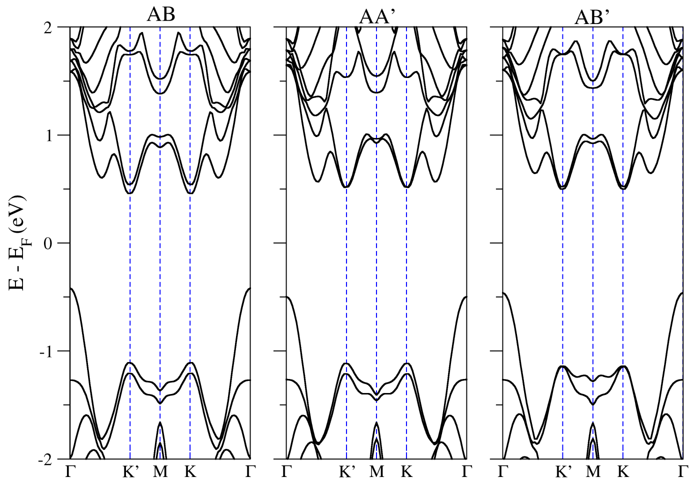

Figure S1. Band structures for the most stable MoS_2 pristine bilayers in the 1×1 unit cell. The Fermi energy is set to 0 eV.

SII Mo-impurities in AB stacking

Because the most stable configuration belongs to the 2H-phase, we discuss the properties of impurity within bilayers focusing on the AA' and AB' stackings in the main text. Another type of stacking labeled as AB is possible, in which a layer just glides on the other layer. Although our results show that pristine AB bilayer is slightly more stable than the pristine AA' bilayer, the presence of Mo_{imp} changes the stability order in the order of eVs. The AB stacking configurations are shown in Fig. S2.

Similar to the 2H-phase, the Mo_{imp} in the 3R-phase prefers an octahedral environment, the T-AB configuration has the lowest energy in the 3R-phase. We next use as energy reference the T-AB' configuration, as in the main text.

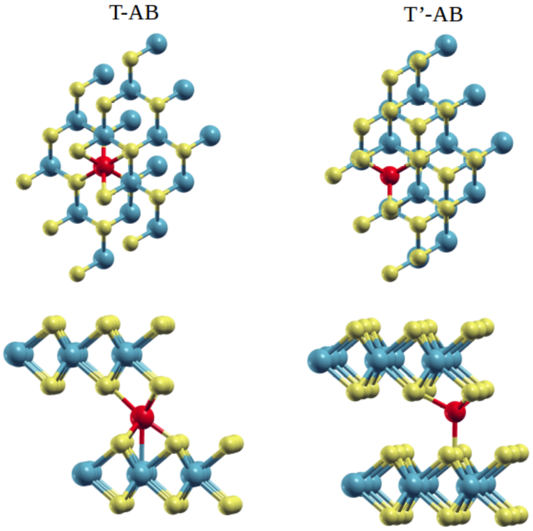

Figure S2. Relaxed structures for the Mo_{imp} in the AB stacking. Red spheres show the Mo_{imp} in each configuration.

The T-AB configuration has a total energy of 0.75 eV in between the T-AB' and H-AA' configurations. The T'-AB configuration with its tetrahedral symmetry lies 1.82 eV above the most stable T-AB', between the T'-AB' and T'-AA' configurations. The total magnetic moment follows a similar trend according to the specific site, 0 for the T-AB configuration and 2 μ_B for the T'-AB configuration.

The band structures presented in Fig. S3 have the same characteristics as in the main text. The T-AB band structure is similar to the one of the ground state (in Fig. 3(a)) and the T'-AB is similar to the T'-AA' (in Fig. 3(c))

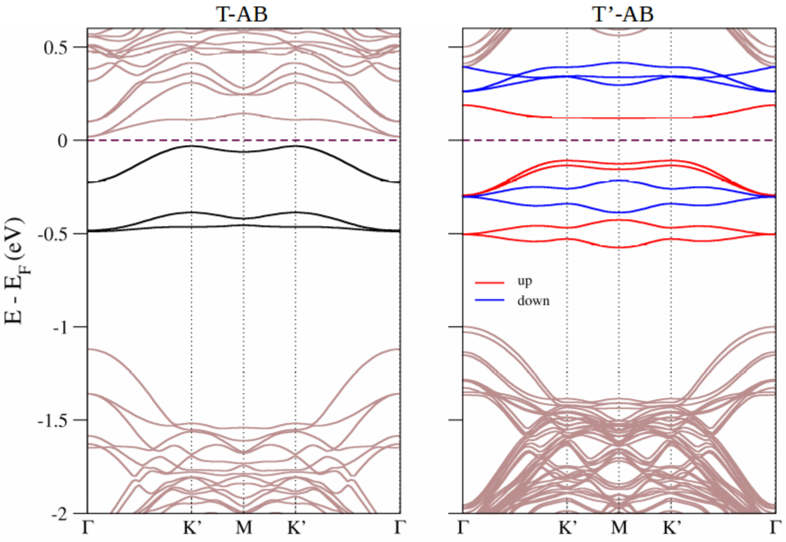

Figure S3. Band structures for the Mo_{imp} in the AB stacking configurations. The Fermi energy is set at 0 eV.

SIII Geometrical parameters

Table S1 collects the geometrical parameters after relaxations. We tabulated the parameters measured near the impurities, labeled with an "*", and those measured far from them. The distance between Mo impurities is about 9.66 Å, corresponding to the magnitude of the lattice vector. The Δ_z and Mo-S distances for pristine stackings are in agreement with previous reported values.[1–4]. The experimental Δ_z is 6.14 Å for the 2H-phase and 6.12 Å for the 3R-phase[5].

Configuration	d*(Mo-S)	d(Mo-S)	Δ_z^*	Δ_z	S-Mo-S angle*	S-Mo-S angle	Layer-gap
Pristine AB	–	2.44	–	6.02	–	81.4	0.88
Pristine AA'	–	2.45	–	6.07	–	81.4	1.02
Pristine AB'	–	2.45	–	6.04	–	81.4	0.93
T-AB'	2.49	2.45	6.07	6.17	80.8	81.3	1.12
T-AB	2.50	2.45	6.21	6.19	80.5	81.7	1.14
H-AA'	2.45	2.45	6.63	6.40	80.7	82.4	1.34
T'-AB	2.52	2.45	6.45	6.45	80.2	81.7	1.42
T'-AA'	2.53	2.45	6.83	6.65	80.6	81.9	1.54

Table S1. Relaxed geometry parameters for MoS_2 pristine bilayers and Mo_{imp} bilayers, given in the order of stability. The length of Mo-S bond is d(Mo-S), and the interlayer Mo-Mo distance is given by Δ_z. The label "*" corresponds to distances measured near the Mo_{imp}. Layer-gap is defined in the main text. All the distances are in Å, angles are in degrees, and energies in eV.

SIV Technical details

Herein we provide extra technical details to assure the reproducibility of the results. The valence electronic configurations for the atoms in the pseudopotentials calculations were $5s^1\ 4d^5$ and $3s^2\ 3p^4$ for Mo and sulfur atoms, respectively. The pseudopotentials core radii and pseudocore radii are included in Table S2.

	r_s	r_p	r_d	r_{pc}
Mo	2.30	2.46	1.67	1.20
S	1.63	1.76	1.94	1.20

Table S2. Pseudopotentials core radii for s, p, d and f channels, and the pseudocore radii r_{pc} for the Mo and S atoms. All the radii are in bhor.

References

1. Ramasubramaniam, A., Naveh, D. & Towe, E. Tunable band gaps in bilayer transition-metal dichalcogenides. *Phys. Rev. B* **84**, 205325 (2011).
2. Liu, K. *et al.* Evolution of interlayer coupling in twisted molybdenum disulfide bilayers. *Nat. Commun.* **5**, 4966 (2014).
3. Kadantsev, E. S. & Hawrylak, P. Electronic structure of a single MoS_2 monolayer. *Solid State Commun.* **152**, 909 (2012).
4. He, J., Hummer, K. & Franchini, C. Stacking effects on the electronic and optical properties of bilayer transition metal dichalcogenides MoS_2, $MoSe_2$, WS_2, and WSe_2. *Phys. Rev. B* **89**, 075409 (2014).
5. Schönfeld, B., Huang, J. & Moss, S. Anisotropic mean-square displacements (MSD) in single-crystals of 2H-and 3R-MoS_2. *Acta Crystallogr. Sect. B: Struct. Sci.* **39**, 404–407 (1983).
